# Prebiotic Chemistry: Geochemical Context and Reaction Screening

**DOI:** 10.3390/life3020331

**Published:** 2013-04-29

**Authors:** Henderson James Cleaves

**Affiliations:** Earth Life Science Institute, Tokyo Institute of Technology, Institute for Advanced Study, Princeton, NJ 08540, USA; E-Mail: cleaves@ias.edu

**Keywords:** chemical evolution, prebiotic organic reactions-prebiotic reactions in the aqueous phase, prebiotic reactions in the solid state, energy sources on the primitive Earth, mineral catalysis

## Abstract

The origin of life on Earth is widely believed to have required the reactions of organic compounds and their self- and/or environmental organization. What those compounds were remains open to debate, as do the environment in and process or processes by which they became organized. Prebiotic chemistry is the systematic organized study of these phenomena. It is difficult to study poorly defined phenomena, and research has focused on producing compounds and structures familiar to contemporary biochemistry, which may or may not have been crucial for the origin of life. Given our ignorance, it may be instructive to explore the extreme regions of known and future investigations of prebiotic chemistry, where reactions fail, that will relate them to or exclude them from plausible environments where they could occur. Come critical parameters which most deserve investigation are discussed.

## 1. Introduction

Prebiotic chemistry is the study of how organic compounds formed and self-organized for the origin of life on Earth and elsewhere [1]. To date a number of proposals have been put forth which might answer some or all of the cogent aspects of this overarching question [2,3,4,5], however all are contingent on a number of as yet unanswered questions. As has been discussed elsewhere [6,7,8], many schemes suffer from a lack of experimental support at crucial junctions, and a lack of concordance with environmental constraints, hence we are left with multiple competing hypotheses.

Several authors (e.g., [9] among others) have suggested the study of the origin of life is separable into historical and ahistorical factors, historical factors being those for which we have no or little remaining evidence, including the geochemical conditions on the Earth’s surface and various contingent factors such as bolide impacts, while ahistorical factors are those less stochastic ones governed by the laws of physics and chemistry. In light of these considerations, Albert Eschenmoser went as far as to state, “Biogenesis, as a problem of science, is lastly going to be a problem of synthesis. The origin of life cannot be “discovered”, it has to be re-invented” [10].

Focusing on the ahistorical processes, those which we can control and test in the lab, criticism has been leveled at the manner in which these processes have been studied [7,11], especially with respect to the manner they attempt to mimic natural environments, as discussed extensively in [12].

## 2. Historical Factors

There is major disagreement in the origins of life research community regarding a number of fundamental questions. Among them are the questions of whether life began on or off-Earth (e.g., on Mars [13], in solar system small body parent bodies e.g., [14], or elsewhere entirely). Limiting the discussion to an Earthly origin, there is a fervent discussion whether it occurred in a surface or sub-surface environment, with submarine hydrothermal environments being the most widely proposed sub-surface venues [15,16,17], and locales such as drying beaches or small evaporative ponds providing a more historically favored alternative [18,19].

**Figure 1:**
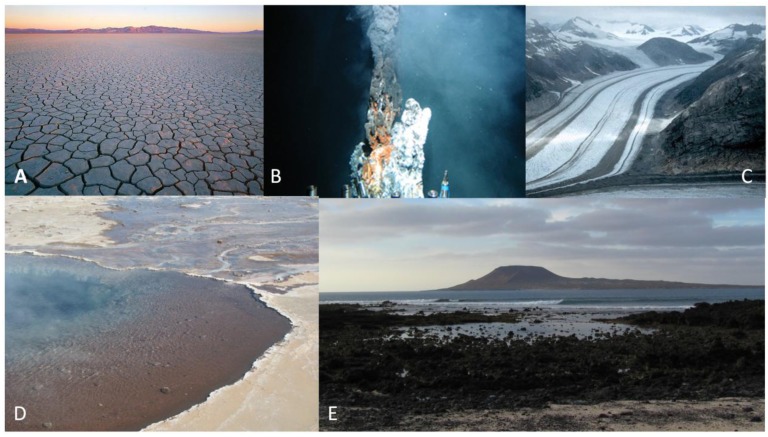
Some possible environments for the origin of life which are still extant on modern Earth. (**A**) Deserts or drying lake beds; (**B**) Deep sea hydrothermal environments; (**C**) Glacial or other icy environments; (**D**) Subaerial hot springs; (**E**) Drying lagoons or beaches.

There may be advantages to aspects of various environments with respect to the chemistry which can easily occur in them, and various extant environments have been considered. Had these environments existed on the primitive Earth, they would be important sites to consider for the origin of life (Figure 1).

There are several models for the origin of life. Opinion is divided as to whether life began with self-replicating nucleic acid molecules (*i.e.*, RNA or RNA like molecules [20,21], self-replicating membranes [22,23], metabolic networks [2,24,25], some combination of these [3,26,27], or perhaps some entirely different organic replicating system [28,29,30,31]. 

It is sometimes assumed that there must be a simple continuity between the environmental context in which life originated and its present state [32], however although this is a parsimonious idea, it ignores the potentially enormous amount of time available between the origin of life and the Last Universal Common Ancestor (LUCA) (see below) as well as the data available from prebiotic organic chemistry. Thus though there may or may not be a “biochemical continuity principle” between the origin of life and contemporary biochemistry [32], there must be a “chemical continuity principle” between geochemical context and the origin of life.

Compounding these uncertainties, very little is known about the conditions which were available or prevalent on the primitive Earth. We do not know, for example, when liquid water, a presumed prerequisite for the origin of life (see for example [33]), first became available, although some tantalizing evidence suggests this may been very early in Earth’s history [34]. We do not know the nature of this water: its temperature, pH or solute content. We also do not know that there was any available sub-aerial land surface, the composition of the prebiotic atmosphere, which has significant implications for climate and the abundance of organics for several reasons [35,36], or indeed much at all conclusively about the types and abundances of the available organic compounds [37,38].

Some of these problems were addressed more than a decade ago with the then current motivation of deciding which type of terrestrial extremophile was the first on Earth [6], and whether one of these might then be indicative in some way of the first organism’s nature and by extension the environment in which it originated. Other authors have noted problems with this same notion [39], and discussion of the dating of LUCA and its nature either point towards an origin of life very similar to present microbial life, or an extended amount of evolution resulting in LUCA (See for example discussions by [40,41,42]). Considerations of the nature of the genetic code point towards the latter interpretation [43], acknowledging that estimation of the tempo of evolution between the origin of life and LUCA may not correspond to later rates of evolution.

**Figure 2:**
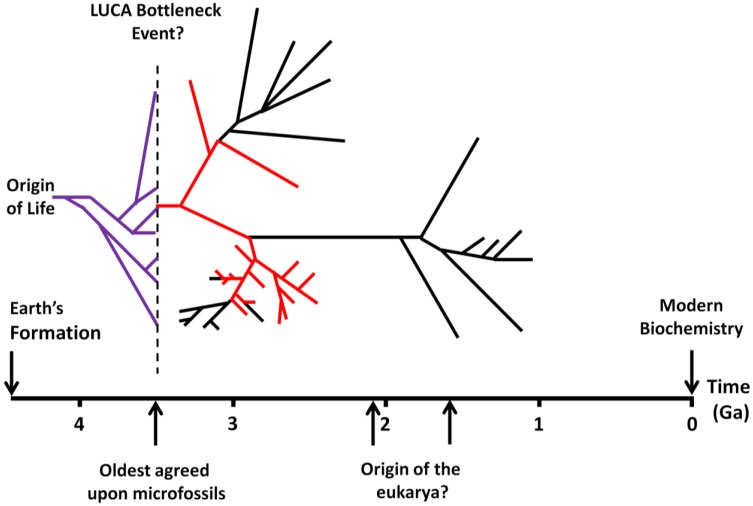
Major events in the history of life on Earth, and the difficulty of pegging events in the evolutionary history of extant organism’s geological timeline and the origin of life. Dating of the origin of the eukarya is taken from [44,56]. Red lines indicate thermophilic or hyperthermophilic lineages and their extrapolation back to LUCA as proposed by [57]. The idea that LUCA was possibly a surviving branch of a previously more diverse ecosystem is represented by the deeper tree shown in lilac. Dating of the origin of life remains unconstrained, and the tree could be stretched or compressed, locally or globally, in time. Lineages to the right of the dashed line should be understood to extend to the present. Figure adapted from [58].

While there have been efforts to calibrate the timing of major branching points in the Tree of Life, they remain open to recalibration, with estimates for the divergence time of the eubacteria and archaebacteria ranging from ~3 to 3.5 Ga, with LUCA possibly dating as far back as 4.3 Ga [44,45] (Figure 2). These questions are not settled, and given the paucity of surviving geochemical evidence of the time period in question [46] and intrinsic error in the techniques used [47], they may not be soon.

## 3. The Tempo and Timing of the Origin of Life

It is unknown when life began on Earth. Evidence has been derived from fossil and isotopic signatures [48,49,50]. It is likewise unknown how life actually began soon after conditions became favorable for the origin of life. Available geochemical evidence leaves open a window of almost 1 Ga, which is an enormous time span in terms of organic chemistry, and regrettably tells us little about the pace of chemical evolution, self-organization and the origin of life. One Ga represents an enormous amount of time in terms of the stability of organic compounds. For example, at 25 °C and ~pH 7, in the absence of complicating factors such as mineral catalysis of decomposition [51,52], this is 9100 half-lives of serine [53], 2.9 × 10^6^ half-lives of cytosine [54], and 1.2 × 10^9^ half-lives of ribose [55].

## 4. Historical Contingency and Immeasurable Variables

What makes origins research so especially difficult is its contingency on immeasurable variables, events which can no longer be observed and which have left little or no trace in the geological record. For example, it is widely believed based on fossil and isotopic evidence that life was present on Earth by 3.5–3.8 Ga [48,49], while physical conditions may have allowed life on Earth as early as 4.4 Ga [34]. Tectonic cycling has erased the record of this period of Earth’s surface conditions to the point where the only viable approach forward, short of recovering terrestrial meteorites from Earth’s moon, are reconstructions from modelers from various fields.

Impact history may be extrapolated from the lunar record, but no “smoking gun” of such an event is left in the terrestrial geological record, and we are not able to confidently choose between exponential decay and catastrophic late-heavy bombardment models [59]. The late heavy bombardment period, which has so fundamentally altered models for the timing of the origin of life [60], rests on evidence collected from the Apollo missions, whose dating and crater association has been reconsidered [61]. Thus, the concept of an ~3.9 Ga planet-sterilizing impact period may also need reconsideration.

Among the crucial environmental variables which thus remain unconstrained, but important for the origin of life, are:

1. **The composition of the early atmosphere.** While it was initially estimated that the early atmosphere was highly reducing, others have argued that it was not, and an atmosphere essentially similar to the present day one, albeit without abundant molecular oxygen was prevalent. This would affect the flux of solar radiation at various wavelengths reaching surface environments [62], the braking rates of impactors [35], as well as the flux and type of organics reaching the surface.
2. **The availability of subaerial landmasses,** which would be important for reactions which rely upon drying or evaporative concentration [19,63].
3. **The composition and properties of global and local primitive water bodies** (e.g., pH, salinity, temperature), which would affect aqueous chemistry in many subtle ways (as discussed below).
4. **Prevailing temperatures on the prebiotic Earth’s surface,** which has fundamental consequences for organic chemical processes (also discussed below).

**Figure 3:**
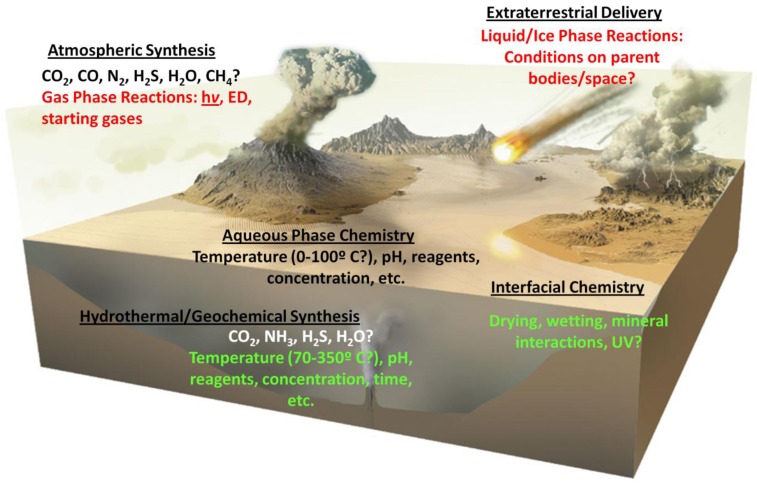
Interconnectedness of environmental conditions with potential early Earth organic chemistry with respect to possible sites for the origin of life. Illustration modified with the permission from [64].

Figure 3 depicts some of the possible environments and organic input sources on the primitive Earth, and important variables which could have affected the chemistry which occurred in them.

## 5. Organic Chemistry as the Critical, Ahistorical Factor

With so many uncertainties remaining in our understanding of the timing and location of the origin of life on Earth, physical organic chemistry, which in many ways overlaps with the purview of prebiotic chemistry, may offer the simplest and most discriminating means of differentiating productive from non-productive models for the origin of life. While it is likely true that life is a process [65], it is undoubtedly true that it is a process manifested by chemical components. 

**Figure 4:**
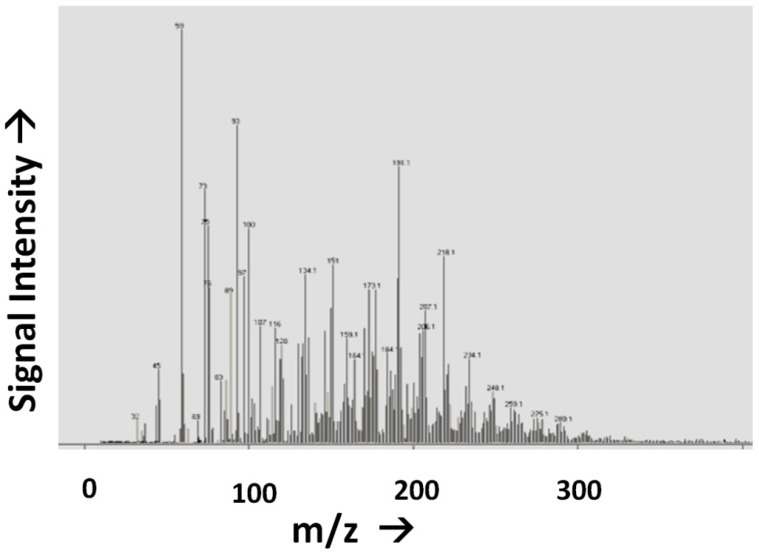
Direct Analysis in Real Time of Flight (DART-ToF) mass spectrum of the reaction products from the reaction of 1 M HCHO with 1 M NH_4_CN. Although this is formally the Strecker synthesis of glycine, clearly a much more complex reaction mixture is generated, in which glycine (m/z MH+ ~76.1) is a relatively minor component.

The behavior of organic compounds is highly dependent on context, and it cannot be assumed that a reaction that has been shown to occur under one set of conditions may therefore be assumed to occur robustly, if at all, under any set of geochemically relevant conditions. The following discussion is not exhaustive, nor is a complete survey of its application in the literature included. Nevertheless, it highlights some major measurable variables, for example:

1. **pH.** A change in the pH value of the reaction environment by single pH unit can easily change reaction rates by a factor of 10. At some pH values reactions may be so inhibited, no observable reaction occurs at all. The implications for prebiotic chemistry are large, and numerous examples of dramatic pH-dependent effects exist in the literature. For example the keto-enol equilibria of the nitrogenous bases in nucleic acids, e.g., [66]; vesicle formation and stability [67], primary synthetic reactions such as the formose reaction and cyanide polymerization [68,69], and the stability of various organic compounds potentially important for the origin of life, including, but not limited to sugars [55], nucleobases [54] and small molecules such as HCN and formamide (HCONH_2_) [70].
2. **Concentration.** Reaction rates, with few exceptions, are often proportional to reactant concentration, since the probability that two molecules will come together and react decreases as the concentration of reactants is lowered. While some multi-reactant processes may proceed in extremely dilute solution, e.g., [71], others are markedly slowed or do not proceed at all upon dilution, e.g., [68,72]. Concentration is also pathway-dependant; compounds must find their way by plausible processes to the environment in which they become concentrated. For example, a considerable body of work has studied the possible role of HCONH_2_ as a precursor to prebiotic organics. To date, reactions have not been shown to work in dilute aqueous solution, and appear to largely require temperatures above the boiling point of water [73]. Concentration also includes two limits, a precipitation limit, beyond which compounds are insoluble, and a dilution limit, beyond which reaction is undetectable. In practice, the analytical limit of detection may be reached, but this can be overcome by various techniques. These are relatively trivial problems compared with the problem of not knowing where reactions become unproductive.
3. **Temperature.** Most reaction rates scale as a function of temperature, according to the well-known Arrhenius equation. A typical organic transformation may increase or decrease in rate by a factor of 2 to 3 for each 10 °C change in temperature. For complex multi-component or multi-pathway reactions, these ratios may be extremely important in determining the course of reactions and their outcomes. In cases where multiple chemical steps occur simultaneously, and there are multiple reactants which are sensitive to temperature effects, the most sensitive component deserves the most careful consideration. While it may be possible to speed reactions by heating them, there may be activation barriers which produce unexpected effects at both higher and lower temperatures. As an example, recent experiments demonstrating increasing efficiency of RNA replicases have resorted to conducting reactions in ice eutectics, owing to the decreased rates of organic degradation at low temperatures [74]. Such experiments also take advantage of the concentrating effects of ice eutectics and the stabilization of weak interactions, such as those mediated by hydrogen bonding, at low temperatures.
4. **Time.** Lazcano and Miller observed that all known prebiotic reactions are fast [75]. Orgel responded by pointing out that our knowledge of prebiotic chemistry may be more constrained by the lengths of post-doctoral fellowships than by any requirements of the chemistry involved [76]. This consideration relates intrinsically to variables of concentration, pH and temperature discussed above. In some cases, it may be almost impossible to produce a laboratory reconstruction of a process for temporal reasons. Extrapolation is perfectly reasonable in such cases, with some degree of caution. To an extent, time is exchanged for temperature in prebiotic simulations. As mentioned above, it is unknown what the average or range of surface temperatures was on the primitive Earth. Micro-environments must also be considered, for example, surface temperatures on the modern Earth range from ~−90 °C to ~+60 °C.
5. **Pressure.** Environments where pressure becomes a significant variable already make some assumptions about other relevant variables, for example temperature. The pH of ambient conditions and the pK_a_ of reactants may change dramatically under extreme pressure conditions, and some remarkable pressure-dependant effects have been observed in prebiotic studies of peptide oligomerization, as might occur in deep marine sediments [77]. It is interesting to speculate how difficulties of working with this parameter may skew our knowledge of abiotic organic chemistry.
6. **Ionic Strength.** Depending on the reaction in question, this may have a relatively minor influence on chemistry, or be a significant factor. Some predictions can be made, but this is certainly worth exploring, for example in the way inorganic solute concentration may limit the ability of processes such as eutectic freezing to concentrate reactive organic solutes [78,79].
7. **Influence of Inorganic Reactants.** Ammonia, sulfide, various transition metals, etc. can significantly affect the course of reactions. Ammonia and sulfide, depending on pH, can be significantly reactive with important prebiotic reactants [80]. It is probably not reasonable, and possibly not productive to explore them all experimentally, and it may irrelevant to do so in some cases, however some merit at least cursory consideration where they might be expected to be relevant. For example, it is well-documented that transition metals can have significant effects on the reactivity of various compounds including amino acids and peptides [52,81]. Simple oxoanions, such as borate and silicate, have also been found to have profound effects on more complex chemistries such as sugar forming reactions [82,83,84].
8. **Reactant Continuity.** Each reactant in any proposed scheme or reaction must fit viably with the above-mentioned considerations. For example, while glyceraldehyde may be a perfectly reasonable reactant, its derivation from a formose like process would inevitably lead to the presence of other carbohydrates and carbohydrate-like compounds [85,86]. These would, in the absence of geochemically viable purification schemes, carry over into subsequent reaction steps. As discussed below, many prebiotic processes produce extraordinarily complex mixtures, and the mere presence of a compound cannot guarantee its ability to react with a second reagent. Even novel syntheses of glyceraldehyde from other processes also result in the presence of numerous other compounds [87].
9. **Reagent Purity.** In almost all known examples of prebiotic synthesis starting from primary reactants such as atmospheric gases, complex mixtures result, in which competing side reactions would be difficult to avoid. For example, in many typical primary syntheses, the yield of glycine is often quite high relative to other measured organic species, though its overall yield is rather low. Glycine represents ~0.01 wt% of the organic content of the Murchison meteorite [88] and is present alongside some thousands or millions of other organic molecule types [37]. The same is true of the yield of glycine from electric discharge experiments, where despite being one of the most abundant single products, it only accounts for ~2% of the input carbon [89], or 0.6% of the input carbon from an aqueous HCN oligomerization reaction [90], in both cases again being accompanied by hundreds or thousands of other organic species (Figure 4).

Glycine, being among the most stable of amino acids with respect to thermal decomposition and hydrolysis [53], and being non-volatile, could reasonably be expected to accumulate in evaporative environments [91]. The simple admixture of a likely congener like glycine to a reaction could shed tremendous light on the plausibility of various prebiotic reactions.

1. **Light.** The flux of solar radiation to the primitive Earths’ surface is somewhat poorly constrained, though the sun’s total luminosity was likely considerably lower [62]. The lack of an ozone layer likely permitted much lower and more energetic wavelength radiation to reach the Earth’s surface, however trivial environmental considerations, such as protection by various dissolved inorganic species, or reactions proceeding in interstitial pore water, could have rendered such considerations essentially moot [92]. Nevertheless, the potentially significant effects of both UV and visible light on prebiotic chemistry remain seriously understudied, underscored by the recent results of Sutherland and colleagues, among other older studies [87,93,94,95].
2. **Minerals.** There are a great number of possible minerals which might have contributed some form of catalysis or concentration of various species [96]. For example, iron oxide minerals have been shown to be catalysts for the both the degradation and synthesis of peptides, as well as the degradation of amino acids [51,52]. It would be unreasonable to attempt to test every possible mineral from the start, but it might be instructive to examine the effects of minerals on reactions once those reactions have been shown to work in their absence. It might be a useful exercise to consider which minerals would be likely to be common in any given environment.
3. **Cycling.** Relatively few studies have taken into account the effects of thermal cycling. Among those that have been reported, one showed the formation of peptides in cycling hydrothermal solutions [97], while another showed the formation of peptides in cycling tidal environments [98]. In both cases is was later suggested that the ultimate product yield is very close to the expected thermodynamic outcome in the absence of cycling [72], as may be expected for a simple reversible process, but the phenomenon likely deserves further consideration.

There are of course other potentially important factors which are not as simply studied. For example, it is generally recognized that flux of energetic particles and radiation radioactive decay processes would have been much higher on the primitive Earth than at present [99]. Given the restrictions on and difficulty of working with radiation sources, this is not a simple variable to routinely explore, but further study could be tremendously informative. 

This short list was composed with aqueous phase chemistry in mind, some of the considerations also apply to gas and solid phase reactions. Any of the above can be important variables in some cases, and at some point even limiting factors. Experiments which demonstrate the synthesis of any given organic compound necessarily do so under a set of fixed conditions, including the above mentioned, sometimes under the assumption of operating within some larger schema for the origin of life. 

As discussed above, there is considerable uncertainty as to the conditions present on the primitive Earth, and there is no reason to believe they were any less heterogeneous than at present, and of course other types no longer present may also have existed. While new discoveries may shed some light on this in the future, it is well within our capability now to understand the chemistry which can operate in any environment.

## 6. The Way Forward?

The point of this discussion, rather than being a review of all things prebiotic, it may be that the way prebiotic chemistry as a study has been pursued is actually hampering progress. Experiments are often optimized to make very specific products under very specific conditions, and having found conditions that work, the experimentation ends. It is often then assumed that if a reaction works under one set of conditions, it will work under any set of conditions, which has led to a proliferation of unproductive schemes in the literature. A very simple methodological change could be very useful.

**Figure 5:**
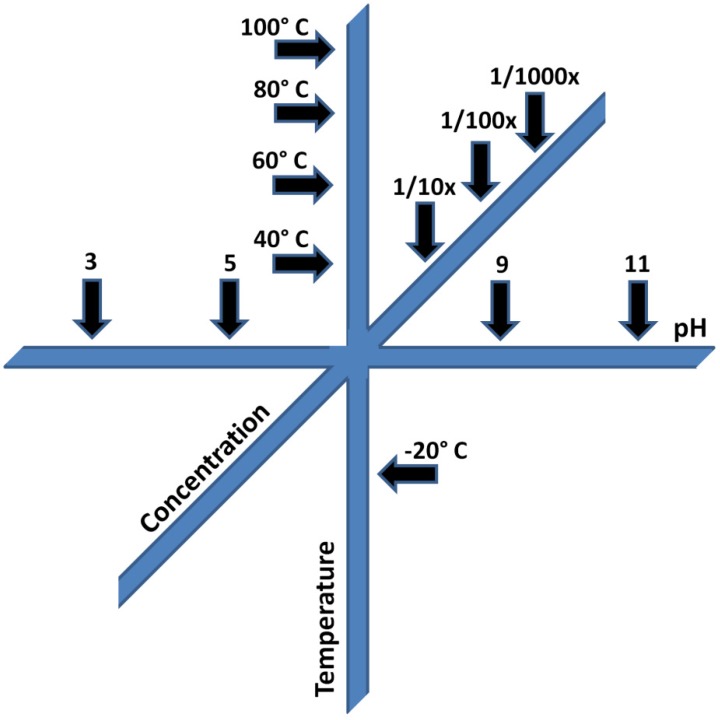
Example of a reaction matrix for screening prebiotic reactions.

It is not suggested that every reaction be tested against every possible range of conditions which could have been available, rather that every reaction should be tested to the point of failure with respect to a few parameters which can be explored simply using a reaction screening matrix (Figure 5).

For example, when conducting microscale test reactions in small test tubes, it is instructive to pick a central pH and reagent concentration value, then prepare a parallel series of reactions in which the pH is changed by 2 pH units in either direction until reaching extremes such as pH 1 and pH 11. Likewise, a simple series extending from the central reaction at temperature increments of, for example, 20 °C, is instructive regarding activation energies the and temperature sensitivity of reactants. A simple serial dilution set of reactions will likewise be extremely informative of reactant concentration dependence.

This will necessarily increase the time it takes to conduct experiments, but perhaps in a manageable way with careful experimental design, and it is worth noting that these are problems which lend themselves especially well to automation and high-throughput screening. The payoff in terms of understanding the reaction parameters is likely to be high, and is crucial for this area of research. 
